# Antimicrobial activities of a small molecule compound II-6s against oral streptococci

**DOI:** 10.1080/20002297.2021.1909917

**Published:** 2021-03-30

**Authors:** Jin Zhang, Xinyi Kuang, Yuanzheng Zhou, Ran Yang, Xuedong Zhou, Xian Peng, Youfu Luo, Xin Xu

**Affiliations:** a State Key Laboratory of Oral Diseases, National Clinical Research Center for Oral Diseases, West China Hospital of Stomatology, Sichuan University, Chengdu, China; b Department of Cariology and Endodontics, West China Hospital of Stomatology, Sichuan University, Chengdu, China; c State Key Laboratory of Biotherapy, West China Hospital, Sichuan University, Chengdu, China; d Department of Pediatric Dentistry, West China Hospital of Stomatology, Sichuan University, Chengdu, China

**Keywords:** Dental caries, oral biofilm, *streptococcus mutans*, antimicrobial agent, small molecule compound, II-6s

## Abstract

**Background**: The side effects of present antimicrobials like chlorhexidine (CHX) and the emergence of drug resistance necessitate the development of alternative agents to control dental caries.

**Aim**: This study developed a novel small molecule, namely II-6s, and investigated its antimicrobial activities against common oral streptococci associated with dental caries.

**Methods**: The susceptibility of streptococci to II-6s was evaluated by the microdilution method, time-kill assay and scanning electron microscopy. The exopolysaccharides, dead/live bacteria and bacterial composition of the II-6s-treated *Streptococcus mutans*/*Streptococcus gordonii*/*Streptococcus sanguinis* 3-species biofilms were analyzed by confocal laser scanning microscopy, fluorescent *in situ* hybridization and quantitative PCR. The anti-demineralization effect and cytotoxicity of II-6s were evaluated by transverse microradiography and CCK-8 assay, respectively. Repeated exposure of *S. mutans* to II-6s was performed to assess if II-6s could induce drug resistance.

**Results**: II-6s exhibited antimicrobial activity similar to CHX against *S. mutans, S. gordonii* and *S. sanguinis* and significantly inhibited exopolysaccharides production, live bacteria and the demineralizing capability of the 3-species streptococcal biofilms. Besides, II-6s showed reduced cytotoxicity relative to CHX and did not induce drug resistance in *S. mutans* after 15 passages.

**Conclusion**: - II-6s may serve as a promising part of a successful caries management plan.

## Introduction

Dental caries is one of the most prevalent non-communicable diseases affecting human beings [1,2]. The oral microbiome maintains a symbiotic relationship with the host in oral health [3]. However, microbial dysbiosis occurs with local environmental changes such as frequent carbohydrate intake and/or reduced saliva flow, which lead to prolonged periods of low pH in the biofilm [3]. Such acidic condition is conducive to the growth of bacteria with an acidogenic and acid-tolerant phenotype, thus increasing the risk of dental caries [4]. Oral streptococci are the dominant species in the oral biofilm [5], and *Streptococcus mutans* is one of numerous cariogenic species due to its acidogenicity and aciduricity. In addition, *S. mutans* can synthesize exopolysaccharides (EPS) which mediate microbial adherence to tooth surface and cell-to-cell adhesion and enhance mechanical stability of biofilms, further contributing to the development of dental caries [3,6–9].

Various measures have been developed to control dental caries, including the daily use of fluoride toothpaste and floss, water fluoridation, dental sealant and antimicrobial mouthwash [10]. Antimicrobials have also shown effectiveness in the reduction of cariogenic biofilm, and thus helping the management of dental caries [11,12]. Chlorhexidine digluconate (CHX) is the most commonly used antimicrobial to control oral biofilm, particularly in high-risk individuals for caries [13–16]. However, this broad-spectrum chemical compound has adverse effects such as bitter taste, discoloration of teeth, mucosal soreness, temporary taste disturbances, hypersensitivity, and potential disturbance of microbial equilibrium within the oral ecosystem [17]. In addition, the long-term application of antimicrobials may induce drug resistance [18–20], further necessitating the development of a novel agent for the control of oral biofilm.

Antimicrobial small molecules are promising for the control of microbial biofilm due to their structural versatility, relatively low cost, and ease of control and use [21,22]. Previous studies have reported various synthetic small molecules which display moderate efficacy against cariogenic bacteria and biofilms [23–25]. Reasonable structural design and scientific screening are important for the development of novel antimicrobial small molecules. In a previous study, we performed a phenotypic screening of a small molecule library against methicillin-resistant *Staphylococcus aureus* (MRSA) and identified NPS-2143, an antagonist of a calcium-sensing receptor, showing bacteriostatic activity against MRSA. We further designed and synthesized a series of derivatives of NPS-2143, and identified an alcohol compound #48, exhibiting decent antimicrobial activity against MRSA and methicillin-susceptible *S. aureus* [26]. The present study aimed to evaluate the antimicrobial activity of compound #48, namely II-6s, on oral streptococci, and to explore its potential use in the control of dental caries. We found that II-6s exhibited good antimicrobial activities against oral streptococci in both planktonic culture and multispecies biofilms, with a lower cytotoxicity relative to CHX. In addition, II-6s significantly reduced the demineralizing capability of streptococcal biofilms, and did not induce drug resistance in *S. mutans* after repeated exposure.

## Materials and methods

### Synthesis and preparation of II-6s

**Figure 1.**
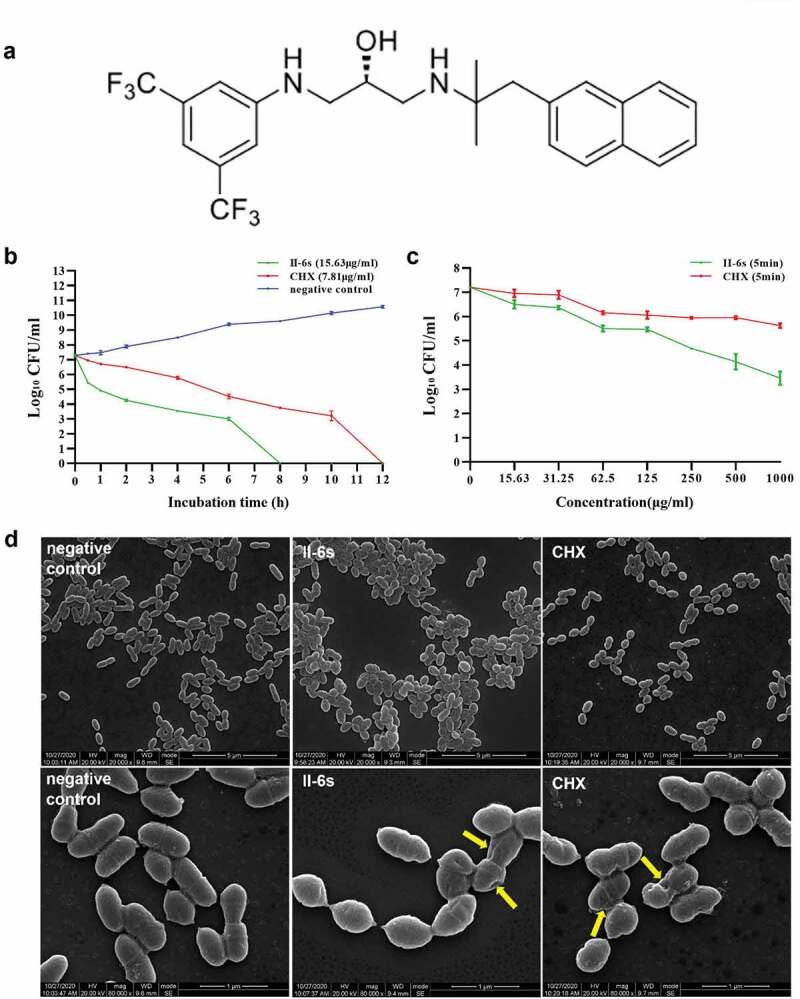
Antimicrobial activities of II-6s against *S. mutans*. (**a**) Chemical structure of the small molecule compound II-6s. (**b**) Time-kill curve of II-6s or CHX against *S. mutans* at MBC. (**c**) Dose-dependent killing curve of II-6s or CHX against *S. mutans* within 5 min. Results are expressed as log_10_ (CFU/ml) and data are presented as means ± SD. (**d**) Scanning electron micrographs of *S. mutans* morphology after treatment with II-6s or CHX at MIC for 12 h. Negative control was untreated *S. mutans*. Yellow arrows indicate that the smooth surface of *S. mutans* cells became uneven and plicated

The synthesis of II-6s was described in our previous study [26]. In short, 3,5-Bis(trifluoromethyl)aniline was reacted with epichlorhydrin and treated with KF to yield an epoxide. Then, the epoxide reacted with solutions of 2-methyl-1-(naphtha-len-2-yl)propan-2-amine in ethanol and the target compound (*S*)-1-((3,5-bis(trifluoromethyl)phenyl)amino)-3-((2-methyl-1-(naphthalen-2-yl)propan-2-yl)amino)propan-2-ol, namely II-6s (Figure 1a) was obtained.

## Bacterial strains and growth conditions

*Streptococcus mutans* UA159, *Streptococcus gordonii* DL1 and *Streptococcus sanguinis* ATCC10556 were obtained from the State Key Laboratory of Oral Diseases (Sichuan University, Chengdu, China). All strains were routinely cultured in brain heart infusion broth (BHI; Difco, Sparks, MD) under anaerobic conditions (5% CO_2_) at 37°C. For biofilm formation, bacteria were cultured in BHI broth supplied with 1% sucrose (designated BHIS).

## Bacterial susceptibility assays

The minimum inhibitory concentration (MIC) and minimum bactericidal concentration (MBC) of II-6s against *S. mutans, S. gordonii*, and *S. sanguinis* were measured by a microdilution method in BHI as described previously [23,27]. The bacterial inoculum for the experiment was adjusted to 1 × 10^7^ colony-forming units (CFU)/ml for *S. mutans, S. gordonii*, and *S. sanguinis* based on the optical density at 600 nm (OD_600_) versus a CFU/ml graph of each bacterium. Bacterial suspension was added to 96-well microtiter plates which were supplemented with serially diluted concentrations of II-6s ranging from 0.98 to 500 μg/ml. Each plate also included CHX ranging from 0.98 to 500 μg/ml as a positive control, test bacteria and BHI broth as a negative control, BHI broth containing equivalent DMSO (0.5% to 0.001%, ν/ν) as a solvent control and a blank control. To determine the MBC, aliquots (50 μl) of all wells with test concentrations equal to or higher than the MIC were inoculated on BHI agar plates (1.5% agar; Difco, Sparks, MD) for 48 h. The lowest concentration of the test solutions that allowed no visible growth on a BHI agar plate was taken as MBC. The experiment was performed in triplicates and repeated at least three times.

## Biofilm susceptibility assays

The effect of II-6s on *S. mutans, S. gordonii*, and *S. sanguinis* biofilm was examined by the microdilution method described previously [28]. *S. mutans, S. gordonii*, and *S. sanguinis* (1 × 10^7^ CFU/ml) respectively, were cultured in BHIS broth and II-6s (0.98 to 500 μg/ml) in 96-well microtiter plates for 24 h. A parallel study was also performed with BHIS as a negative control. After incubation, the culture supernatant and agent were removed, and each well was gently washed three times with phosphate-buffered saline (PBS), then fixed the adherent biofilm was fixed with methanol for 15 min, and stained with 0.1% (wt/vol) crystal violet (Sigma) for 5 min. The excess crystal violet stain in each well was rinsed away with PBS until the blank control wells were colorless. Finally, 200 μl of 95% ethanol was added to each well to dissolve the crystal violet attached to the biofilm. The plate was shaken at room temperature for 30 min, and the absorbance at 595 nm was recorded. To examine the effects of II-6s on mature biofilm, bacterial suspensions were inoculated into a 96-well microtiter plate containing BHIS and incubated at 37°C for 24 h. After biofilm developed, the supernatant from each well was decanted and planktonic cells were removed by washing with sterile PBS. Fresh BHI broth containing II-6s (0.98 to 500 μg/ml) was added to the one-day biofilm, and the plate was further incubated for 24 h. The control wells contained BHIS broth without II-6s. Similarly, the biofilm was fixed, stained, washed, and quantified as described above. The minimum biofilm inhibitory concentration (MBIC) was the lowest concentration of II-6s that showed at least 90% inhibition of biofilm formation, and the minimum biofilm reduction concentration (MBRC) was defined as the lowest concentration of II-6s that resulted in at least 90% reduction of biofilms compared with that in the negative control. The experiment was performed in triplicates and repeated at least three times.

## Time-dependent and dose-dependent killing assays

The time- and dose-dependent killing capacities of II-6s were assessed by the method modified from that of Koo et al. [29]. *S. mutans* was diluted to 1 × 10^7^ CFU/ml in BHI broth containing test compounds or not, and incubated in 5% CO_2_ at 37°C. For the time-kill curve, the final concentration of II-6s and CHX was equivalent to their MBC. At 0, 0.5, 1, 2, 4, 6, 8 and 10 h, samples were serially diluted in PBS and 50 μl aliquots were spread onto BHI agar medium. For the dose-dependent killing curve, *S. mutans* was treated with different doses of II-6s and CHX for 5 min before diluted and spread. All plates were incubated anaerobically for 48 h followed by enumeration of CFU. The time-kill or dose-dependent killing curves were constructed by plotting the log_10_(CFU/ml) versus the incubation time or concentration. The experiment was performed in triplicates and repeated at least three times.

## Observation of bacterial morphology by scanning electron microscopy (SEM)

*S. mutans* was cultured in BHI (treated with II-6s or CHX at their MIC) at 37°C for 12 h, while the bacterial cells without treatment were used as the negative control. Then, all samples were centrifuged at 5,000 rpm for 3 min at 4°C and washed three times with PBS. The bacterial cells were fixed in 2.5% glutaraldehyde at 4°C for more than 4 h and dehydrated in 30, 50, 70, 80, 85, 90, 95 and 100% ethanol. All specimens were sputter-coated with gold and observed via SEM (FEI, Hillsboro, OR, USA) working in a secondary-electron mode with a high voltage condition (20 kV) and a working distance of 9.7 mm. All images were taken from randomly selected positions at 20,000 × or 80,000 × magnification.

## Biofilm imaging

To form 3-species biofilms, bacterial suspensions were mixed to obtain an inoculum consisting of *S. mutans, S. gordonii*, and *S. sanguinis* (1 × 10^5^ CFU/ml for each strain, inoculum ratio = 1:1:1) in BHIS broth as described previously [30]. The inoculum (250 μl) was added into the chemotaxis chamber (μ-slide 8well, 80,826; Ibidi) and incubated for 24 h. After the multispecies biofilms were formed, they were treated with different concentrations of II-6s, CHX and PBS for 5 days (5 min, three times per day).

For EPS staining, 2.5 μM Alexa Fluor 647-labeled dextran conjugate (Molecular Probes) was added to the culture medium from the beginning of the biofilm formation, and the bacteria were stained with 2.5 μM SYTO 9 (Molecular Probes, Invitrogen) for 15 min after biofilms formed [31]. The biofilms were imaged with a DMIRE2 confocal laser scanning microscope (Leica, Wetzlar, Germany) equipped with a 60 × oil immersion lens.

For dead/live imaging, multispecies biofilms were stained with the Live/Dead BacLight Bacterial Viability Kit (Molecular Probes, Invitrogen), and the reagents were applied to the biofilms at final concentrations of 2.5 μM SYTO 9 and 2.5 μM propidium iodide for 15 min. Live/Dead staining is a measure of membrane damage since SYTO 9 penetrates intact cytoplasmic membranes, while propidium iodide is only able to penetrate damaged membranes. The dyed biofilms were imaged with a Leica DMIRE2 confocal laser scanning microscope as in EPS staining.

For fluorescent *in situ* hybridization imaging, biofilms were fixed in 4% paraformaldehyde overnight and investigated by species-specific probes (Table S1) as described previously [30–32]. In brief, prehybridization (15 min, 46°C) was performed in 250 μl hybridization buffer with 20% formamide in the absence of any oligonucleotide probes. The incubation time for the hybridization was almost 90 min at 46°C in the dark. After the incubation, biofilms were transferred into washing buffer preheated to 48°C and incubated for 15 min at this temperature. After staining, the biofilms were imaged with a Leica DMIRE2 confocal laser scanning microscope as in EPS staining.

Each biofilm was scanned at five randomly selected positions and the experiments were performed in triplicates on three independent occasions. All three-dimensional reconstructions of the biofilms were processed with Imaris, version 7.2.3 (Bitplane, Zürich, Switzerland), and the quantification of fluorescence levels was performed with ImageJ (with the plugin COMSTAT) (National Institutes of Health, Bethesda, MD).

## DNA isolation and real-time PCR

Total DNA of biofilms was isolated and purified using a TIANamp Bacteria DNA kit (Tiangen, Beijing, China). The bacteria were lysed using enzymatic lysis buffer (20 mM Tris-HCl, pH 8.0, 2 mM sodium EDTA, and 1.2% Triton X-100) containing 25 mg/ml of lysozyme at 37°C for 1.5 h. The purity and concentration of DNA were determined by a NanoDrop 2000 spectrophotometer (Thermo Scientific, Waltham, MA, USA). The extracts were placed at −20°C before use. TaqMan real-time PCR (Life Technologies, Carlsbad, CA, USA) was applied to quantify the absolute number of *S. mutans, S. gordonii* and *S. sanguinis* as described by the manufacturer (TaKaRa, Dalian, China). The sequences of primers (Table S2) were in accordance with previous studies [33,34]. The standard curves of these bacteria were plotted using cycle values of DNA, which stands for corresponding concentrations of bacteria from 1 × 10^8^ CFU/ml to 1 × 10^4^ CFU/ml (Figure S1). The number of each strain within 3-species biofilms was calculated based on standard curves. The experiment was conducted in triplicates and repeated three times.

## Transverse microradiography

The labial dental crown of bovine incisors was cut into 5 mm × 5 mm × 4 mm by a diamond-coated band saw with continuous water cooling (Struers Minitom; Struers, Copenhagen, Denmark). These bovine specimens were embedded in polymethylmethacrylate and labial enamel surfaces were ground flat with water-cooled carborundum discs of waterproof silicon carbide paper (Struers) of various grits (1,000, 1,200, 2,400, 3,000 and 4,000). Then, enamel blocks were painted with two layers of acid-resistant nail varnish, leaving a 4 × 4 mm window exposed on the labial enamel surface. These specimens were measured by the Rockwell hardness test [35] (Zwick, Ulm, Germany) and those in the range from 314.6 to 382.4 Knoop Hardness Numbers were selected for the demineralization assessment. All the polished samples were sonicated in distilled water for 5 min to remove the residual abrasives and sterilized in an ethylene oxide sterilizer (AnproleneAN 74i, Andersen, Haw River, NC, USA).

An amount of 2 ml bacterial suspension solution contained *S. mutans, S. gordonii* and *S. sanguinis* (1 × 10^7^ CFU/ml for each bacterium) was added to each well of a 12-well plate with a bovine sample, which was cultured anaerobically in a BHIS medium at 37°C (5% CO_2_) for 24 h. The specimens were treated with PBS, 0.2% of CHX or different concentrations of II-6s (15.63 to 125 μg/ml) for 5 days (5 min, three times per day). The pH of all experimental solutions was adjusted to 7.0 before treatment. All specimens were washed with PBS and refreshed with BHIS after every exposure. After 5 days of incubation, samples were rinsed with distilled water to remove the remaining biofilms, then cut again and polished with waterproof silicon carbide abrasive papers (800–4,000 grit; Struers, Copenhagen, Denmark) to a thickness around 100 μm [36]. X-ray films of each slices were acquired by an X-ray generator (Softex, Japan) equipped with a microradiography camera and then were further examined using a Zeiss AXIO Imager A2 microscope (Carl Zeiss, Germany). Quantitative data of mineral loss and lesion depth were acquired by a calibrated analysis system TMR2006 (Inspektor Research Systems BV, Netherlands). Data were obtained as the means of nine separate samples.

## *In vitro* cytotoxicity assay

The cytotoxicity of II-6s was evaluated by the Cell Counting Kit-8 (CCK-8; Dojindo, Kumamoto, Japan) assay as described by Park et al. [37]. Human gingival epithelial cells (HGE), human oral keratinocytes (HOK) and macrophages RAW264.7 (RAW264.7) were provided by the State Key Laboratory of Oral Diseases, Sichuan University, China. HGE, HOK and RAW264.7 were grown in Dulbecco’s modified Eagle’s medium (DMEM; GibcoTM, Invitrogen, Carlsbad, CA, USA) supplemented with 10% fetal bovine serum (FBS; Invitrogen) and 1% penicillin–streptomycin solution (Invitrogen) and cultured at 37°C in a humidified atmosphere containing 5% CO_2_. All cells were inoculated into 96 well plates at a density of 1 × 10^4^ cells per well for 24 h and then treated with II-6s (0.98 to 500 μg/ml) for 5 min or 24 h. The positive control was treated with CHX (0.98 to 500 μg/ml) at the same time. After treatment, the wells were washed with sterile PBS and fresh medium was added. 10 μl of CCK-8 solution (10 μl) was added to each well and incubated for 1.5 h. The absorbance of each well was measured at the wavelength of 450 nm against a blank which contained medium only. Cell viability was calculated according to the following formula: percent viability = (A_450_ of treated group − A_450_ of blank control)/(A_450_ of negative control − A_450_ of blank control) × 100%. The experiment was conducted in triplicates and repeated three times.

## Bacterial drug resistance assay

To examine whether *S. mutans* could develop drug resistance against II-6s, MIC measurements after repeated successive passages were performed to analyze the antimicrobial resistance, following a previous method [38]. The MIC values of II-6s against *S. mutans* were tested as described above. After the initial MIC was determined, 20 µl of bacterial suspension in a well showing 1/2 MIC was mixed with 1,980 µl of BHI medium to eliminate the influence of residual drug. A volume of 50 µl of the resultant bacterial suspension was then inoculated onto a BHI agar plate followed by incubation at 37°C for 48 h. The colonies grown on the agar plate were again suspended in BHI broth. The overnight bacterial suspensions were diluted to a concentration of 1 × 10^7^ CFU/ml for the next MIC test. The same procedure was repeatedly performed for 15 passages. An increase of four-fold or higher in MIC over the initial MIC was considered to be the standard for inducing resistance to an antibacterial agent [39]. The assay was done in triplicates and repeated three times.

## Statistical analysis

All experiments were performed in triplicates and repeated at least three times independently. As the normality (checked by the Shapiro-Wilk test) and the equal variance assumptions (checked by the Levene test) of the data appeared to be valid, one-way analysis of variance and post hoc Student-Newman-Keuls test were performed to examine the effect of different treatment groups. The data are presented as the mean ± standard deviation. Data were considered significantly different when the *P* value was <0.05. Statistical analysis of data was performed with the SPSS software, version 16.0 (SPSS Inc., Chicago, IL, USA).

## Results

**II-6s exhibits antibacterial activity similar to CHX against planktonic cells and biofilms of oral streptococci.**

The antimicrobial activities of II-6s on planktonic bacteria and biofilms of *S. mutans, S. gordonii* and *S. sanguinis* are shown in (Table 1). II-6s exhibited comparable antimicrobial activities to CHX. Specifically, the minimum concentrations of II-6s required to inhibit or to kill planktonic streptococcal bacteria ranged from 1.95 μg/ml to 15.63 μg/ml. The MBICs of II-6s against *S. mutans, S. gordonii* and *S. sanguinis* biofilms were 3.91 μg/ml, and the MBRCs of II-6s against *S. mutans, S. gordonii* and *S. sanguinis* biofilms ranged from 3.91 μg/ml to 62.5 μg/ml.

**Table 1.** Antimicrobial effect of II-6s on planktonic cultures and biofilms of *S. mutans, S. gordonii*, and *S. sanguinis* in BHI medium

Bacterial species	II-6s (μg/ml)				CHX (μg/ml)			
	MIC	MBC	MBIC	MBRC	MIC	MBC	MBIC	MBRC
*S. mutans*	3.91	15.63	3.91	62.50	1.95	7.81	0.98	62.50
*S. gordonii*	3.91	15.63	3.91	3.91	3.91	7.81	3.91	15.63
*S. sanguinis*	1.95	7.81	3.91	3.91	0.49	3.91	1.95	3.91

MIC, minimum inhibitory concentration; MBC, minimum bactericidal concentration; MBIC, minimum biofilm inhibition concentration; MBRC, minimum biofilm reduction concentration.

Time-kill assay was further performed to evaluate the kinetic-killing effect of II-6s against the cariogenic *S. mutans*. II-6s at MBC exhibited a time-dependent bactericidal activity against *S. mutans*, resulting in an approximately 3-log_10_ CFU/ml reduction within 2 h. Besides, II-6s displayed a faster bactericidal effect relative to CHX, as no visible colonies were observed on the plate after treatment with II-6s for 8 h, while CHX at the MBC level took 12 h to achieve the same effect (Figure 1b). A dose-dependent killing assay was further performed to compare the bactericidal effect of II-6s and CHX. As shown in the (Figure 1c), II-6s displayed better bactericidal activities than CHX at the equivalent doses after 5-min exposure.

The influence of II-6s on the morphology of *S. mutans* was further evaluated by SEM. As shown in (Figure 1d), untreated *S. mutans* cells were elliptical and regular with a smooth intact surface without apparent cytolysis. *S. mutans* cells treated with II-6s at MIC were deformed and destructed with uneven and crumpled surface. Cells treated with CHX at the MIC level were also wrinkled and even ruptured.

## II-6s inhibits the development and demineralizing capability of 3-species streptococcal biofilms

**Figure 2.**
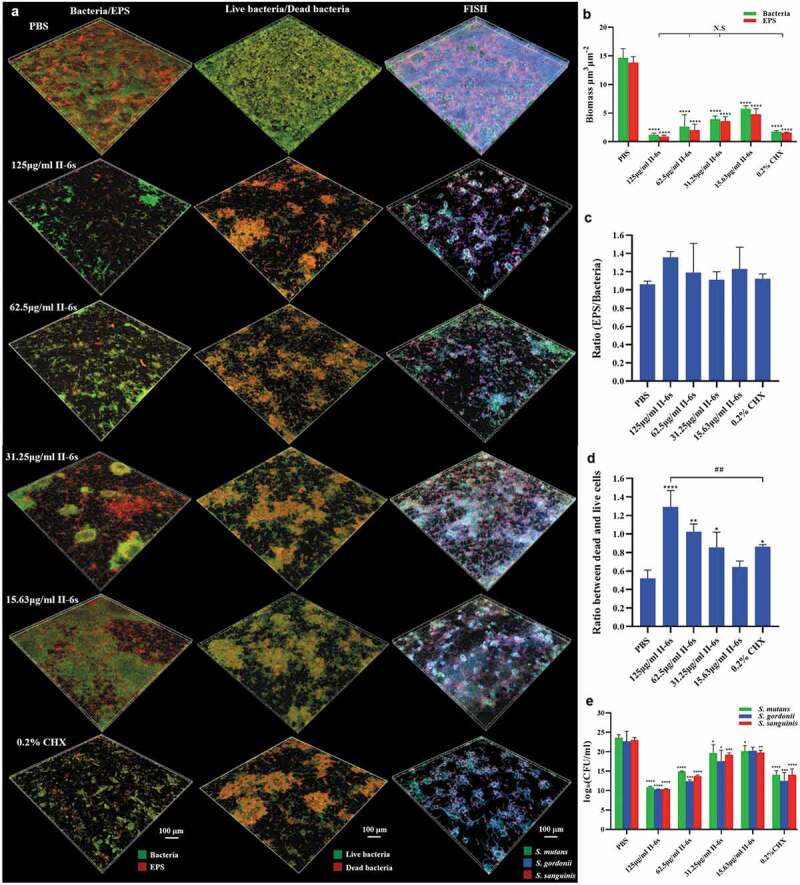
Effect of II-6s on the microbial composition of mixed biofilms. (**a**) Representative images of bacteria/EPS staining, dead/live bacteria staining and fluorescent *in situ* hybridization (FISH) of 3-species biofilms. (**b**) The volume of EPS and bacteria within the biofilms. (**c**) The EPS/bacteria ratio within the biofilms. (**d**) Quantitative analysis of the dead/live bacteria ratio within the biofilms. (**e**) Quantitative analysis of the bacterial species composition based on quantitative PCR. Data are presented as means ± SD. *P < 0.05, **P < 0.01, ***P < 0.001, ****P < 0.0001 as compared to the PBS treated control. ##*P* < 0.01, as compared to the CHX-treated group. N.S., not significant

The development of oral streptococcal biofilms is closely related to bacterial accumulation and EPS production. The effects of II-6s on bacterial cell viability and EPS generation were further assessed within 3-species biofilms. The treatment of II-6s and CHX significantly disrupted the microstructure of 3-species biofilms (Figure 2a). Specifically, the bacterial biomass and EPS production within the biofilms were decreased with the increase of II-6s concentration as compared to the non-treated controls. II-6s at doses ranging from 31.25 μg/ml to 125 μg/ml exhibited equivalent ability to reduce bacterial biomass and EPS production as compared to 0.2% of CHX (Figure 2b). However, neither II-6s nor CHX treatment altered the EPS/bacteria ratio of the biofilms (Figure 2c), indicating that the EPS decrease was parallel to the bacterial eradication in the biofilms. The dead/live bacteria ratios of biofilms treated with II-6s ranging from 31.25 μg/ml to 125 μg/ml were significantly higher than that of the non-treated control, and the dead/live bacteria ratio of biofilm treated with 125 μg/ml of II-6s was even higher than that treated with 0.2% of CHX (Figure 2d). Fluorescent *in situ* hybridization and quantitative PCR were further employed to analyze the microbial composition within 3-species biofilms. II-6s effectively reduced the total biomass of streptococci but did not specifically reduce the cariogenic *S. mutans* in the 3-species biofilms (Figure 2e).

**Figure 3.**
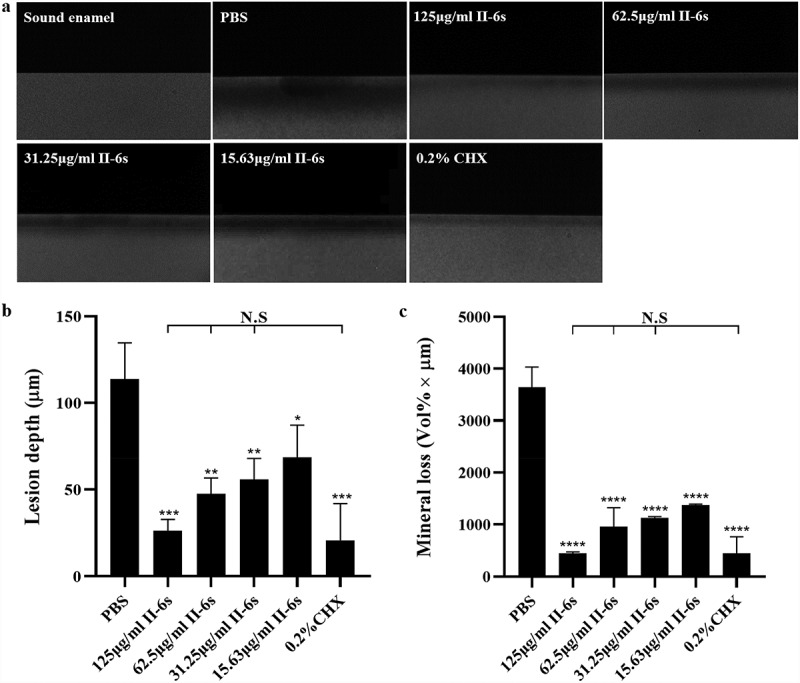
The anti-demineralization effect of II-6s against three-species biofilms. (**a**) Representative transverse microradiography images of bovine enamel slabs exposed to three-species biofilm-induced experimental demineralization. The high-density regions represent the sound enamel tissues, while the low-density shadows indicate the caries-like lesions. (**b**) Lesion depth and (**c**) mineral loss of enamel slabs were calculated. Data are presented as means ± SD. *P < 0.05, **P < 0.01, ***P < 0.001, ****P < 0.0001 as compared to the PBS treated control. N.S., not significant

To further evaluate the inhibitory effect of II-6s on the cariogenicity of the 3-species biofilms, we used transverse microradiography to quantify the biofilm-induced demineralization on bovine enamel slabs. Both II-6s and CHX treatment significantly reduced the demineralization of enamel slabs by streptococcal biofilms, as reflected by decreased lesion depth and mineral loss of enamel slabs. II-6s ranging from 31.25 μg/ml to 125 μg/ml exhibited equivalent inhibitory effect on the biofilm-induced demineralization as compared to 0.2% of CHX (Figure 3).

## II-6s shows lessened cytotoxicity against human oral cells as compared to chlorhexidine

**Figure 4.**
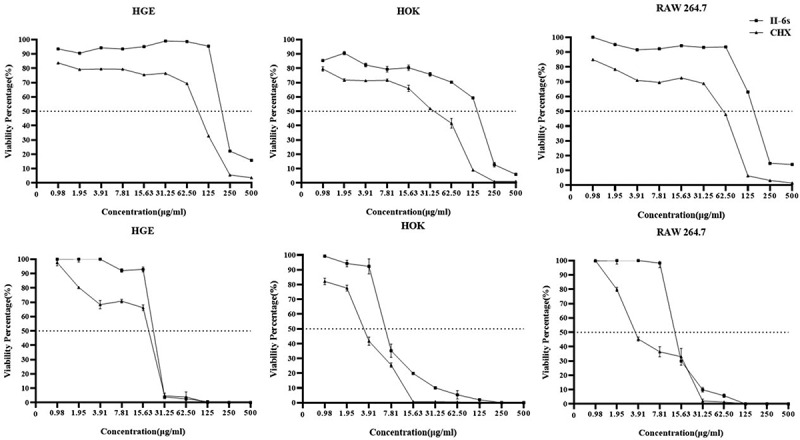
Cytotoxicity of II-6s on human oral cells. The cytotoxicity of II-6s on human gingival epithelial cells (HGE), human oral keratinocytes (HOK), and macrophages RAW264.7 after treatment at indicated concentrations for 5 min (**a, b, c**) and 24 h (**d, e, f**), respectively. Data are represented as means ± SD

The cytotoxicity of II-6s against HGE, HOK and RAW264.7 was assessed after the cells were exposed to the treatment for a duration of 5 min and 24 h, respectively. II-6s showed lessened short-term (5 min) or long-term (24 h) cytotoxicity against the tested cells as compared to CHX. More importantly, the 50% inhibitory concentration (IC_50_) of II-6s on HGE, HOK and RAW264.7 (IC_50_ > 125 μg/ml) for 5 min was much higher than the concentration required to reduce oral streptococcal biofilms (MBRC ranging from 3.91 μg/ml to 62.5 μg/ml). The IC_50_ values of II-6s on HGE, HOK and RAW264.7 in an exposure duration of 24 h were also higher than that of CHX (Figure 4).

## Repeated exposures to II-6s does not induce drug resistance towards *S. mutans*

**Figure 5.**
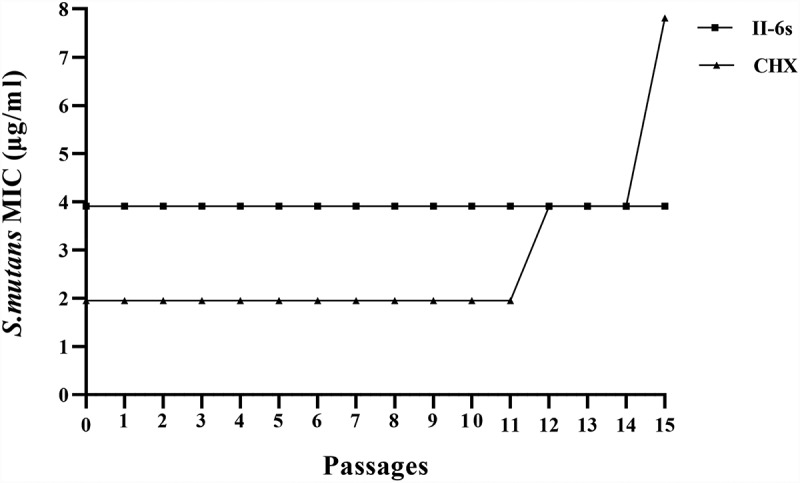
MICs of II-6s and CHX against planktonic *S. mutans* after repeated exposure from passages 0 to 15. Data are presented as mean values of MICs from three independent repeats

The MICs obtained during passaging planktonic *S. mutans* in sub-inhibitory concentrations of II-6s are plotted in (Figure 5). The MICs of II-6s against *S. mutans* were consistent at 3.91 μg/ml and did not change from passages 0 to 15, indicating that repeated exposures of *S. mutans* to II-6s did not induce drug resistance. However, the MICs of CHX increased by four times at the 15th passage.

## Discussion

Dental caries is a prevalent non-communicable disease associated with dental plaque biofilm [1,2]. Effective biofilm control is the prerequisite for the management of dental caries. Chemical methods such as antimicrobial mouth rinses have been recommended as an adjuvant to inadequate mechanical removal of plaque biofilms for high caries-risk individuals [14,15,40,41]. Many antimicrobial agents have shown effectiveness in the control of oral biofilms, such as CHX and cetylpyridinium chloride [35,42–44]. A clinical trial conducted by Rechmann et al. found that approximately 70% of patients at high caries risk were reduced to moderate or low risk in 18 months, when 0.12% CHX was used daily for a week, and repeated every month. This clearly demonstrated the effectiveness of CHX as part of a caries management plan for high-caries risk individuals [41]. However, concerns with these agents were the emergence of microbial resistance and undesirable side effects, such as high cytotoxicity and tooth staining [43–46]. Hence, it is necessary to develop novel antimicrobials to control plaque biofilms. The current study developed a novel small molecule, II-6s, which showed potent antimicrobial effect but with low cytotoxicity, and it did not induce drug resistance in cariogenic *S. mutans*, representing a promising agent that could be used for the control of plaque biofilms.

CHX is currently the most commonly used antimicrobial agent to control plaque biofilms [13]. This broad-spectrum antiseptic has shown effectiveness against a wide variety of oral pathogens *in vitro*, including *S. mutans, Porphyromonas gingivalis* and *Candida albicans*, etc [42,47]. Clinical studies have demonstrated the beneficial activities of CHX in reducing dental plaque biofilms [48,49] and decreasing the incidence of tooth caries among high-risk populations [14–16]. However, CHX is less effective against bacteria deep in the biofilms due to its limited penetration, which is related to its high molecular weight and the fact that cationic CHX molecules potentially interact with matrix components [50,51]. Besides, CHX can cause tooth staining accompanied with unpleasant taste [17]. More importantly, CHX exhibited dose-dependent toxicity on host cells including human gingival fibroblasts, human periodontal ligament cells and human alveolar bone cells [52–54], and it could potentially induce phenotypic adaptation in oral bacteria after long-term application [18,51]. Small molecules with high structural versatility can specifically target cariogenic biofilms by interfering with glucosyltransferases or signaling pathways, usually with less cytotoxicity [24,25,55,56]. Small molecules can even slow down the development of resistance via reduced selective pressures exerted by non-bactericidal mechanisms [57]. In the present study, small molecule II-6s exhibited comparable antibacterial activity with CHX against oral streptococci in either planktonic culture or biofilms, and more importantly, it showed a superior time-dependent and dose-dependent bactericidal effect as compared to CHX. In addition, our SEM data showed that II-6s caused noticeable destruction to the cell membrane of *S. mutans*. The morphology of the damaged cells was similar to those treated with CHX, which is known to exert its antibacterial action via membrane damage, suggesting that II-6s may also function via a similar mechanism. Future studies are warranted to verify the exact mechanism and to investigate whether II-6s could also affect other caries-associated species such as *Lactobacillus* spp., *Bifidobacterium* spp., and *Scardovia* spp. via the same mechanism.

Antimicrobial resistance has become a growing threat to global public health [58]. The antimicrobial susceptibility of oral bacteria can be a decisive factor in the clinical success of anti-biofilm drug regimens [59]. Many isolated oral pathogens like *P. gingivalis* and *Enterococcus faecalis* have exhibited elevated tolerance toward CHX [18,19]. In addition, low-level exposure to CHX, which likely occurs in the deep layers of biofilms, could lead to the development of cross-resistance toward other antibiotics, challenging the effectiveness of biofilm control [60,61]. Our study also found that repeated treatment with CHX could decrease the susceptibility of *S. mutans*. However, the susceptibility of *S. mutans* to II-6s was consistent after repeated exposure for 15 passages, indicating the translational potential of II-6s as a daily used antimicrobial mouth rinse for the control of plaque biofilms.

The cariogenicity of *S. mutans* is largely attributed to the production of EPS [7,62,63]. EPS provides a supporting scaffold to facilitate adherence and accumulation of streptococci and other microorganisms [63], and it have been recognized as the crucial virulence factors contributing to the pathogenesis of dental caries [62]. Inhibition of EPS could disaggregate bacteria and disrupt acidic microenvironments of biofilms [64,65]. Several small molecule compounds have been reported to reduce the accumulation of biofilms on teeth and decrease the occurrence of caries *in vivo* by suppressing EPS synthesis [24,29,65,66].The current study found that II-6s can significantly reduce EPS generation of the streptococcal biofilms, and more importantly, it effectively suppressed biofilm-induced demineralization on tooth enamel, suggesting its potential application in the management of dental caries.

The biocompatibility of novel synthetic compound is a key to clinical application. Gingival epithelium and macrophages are natural barriers to protect the oral mucosa against latent invasion, and keratinocytes are vital to oral mucosa renewal [67–69]. The present study evaluated the cytotoxicity of II-6s against HGE, HOK and macrophages. Our findings revealed that II-6s exhibited less cytotoxicity but comparable antimicrobial efficacy as compared to CHX. The IC_50_ of II-6s on human oral cells was much higher than its antimicrobial concentration, suggesting its safety when used as a topical antimicrobial mouth rinse. Since the stratified epithelial layers of the oral mucosa are regenerating at a high rate and providing protection against mechanical and chemical damage [70], it is conceivable that topical application of II-6s would not impair tissue integrity.

Some cautions should be taken when interpreting data from the current study. Dental caries is closely associated with the microbial ecology of the plaque biofilms [3]. Given the diversity of oral microbiota, whether II-6s treatment could rescue the microbial dysbiosis during caries needs further study with more sophisticated biofilm models. In addition, although the current study demonstrated that II-6s significantly halted the enamel demineralization by the 3-species streptococcal biofilms, further study with dental plaque and caries animal models are still needed to comprehensively evaluate its anti-caries activity.

In conclusion, this study found an antibacterial, low-cytotoxic small molecule compound II-6s, which effectively reduced the EPS production, live bacteria biomass and demineralizing capability of oral streptococcal biofilms, without inducing drug resistance of cariogenic *S. mutans*. This antibacterial compound may serve as a promising part of a successful caries management plan.

## Supplementary Material

Supplemental Material
