# Supplementary material for: Antimicrobial activities of a small molecule compound II-6s against oral streptococci
Source: J Oral Microbiol. 2021 Mar 30;13(1):1909917. doi: 10.1080/20002297.2021.1909917 (PMC8018465; doi:10.1080/20002297.2021.1909917)
Supplement: Supplemental Material [file ZJOM_A_1909917_SM5465.doc]

**Antimicrobial activities of a small molecule compound II-6s against oral Streptococci**

**Jin Zhang, ^a, b^ Xinyi Kuang, ^a, b^ Yuanzheng Zhou,^c^ Ran Yang, ^d^ Xuedong Zhou,^a, b^ Xian Peng,^a^ Youfu Luo, ^c^** **Xin Xu^a, b^***

^a^ State Key Laboratory of Oral Diseases, National Clinical Research Center for Oral Diseases, West China Hospital of Stomatology, Sichuan University, Chengdu, China

^b^ Department of Cariology and Endodontics, West China Hospital of Stomatology, Sichuan University, Chengdu, China

^c^ State Key Laboratory of Biotherapy, West China Hospital, Sichuan University, Chengdu, China

^d^ Department of Pediatric Dentistry, West China Hospital of Stomatology, Sichuan University, Chengdu, China

**Table S1.** Oligonucleotide probes used in fluorescence *in situ* hybridization

| Bacterial species | Sequence (5´- 3´) | References |
| --- | --- | --- |
| *S. mutans* | ACTCCAGACTTTCCTGAC | [1] |
| *S. gordonii* | ACTGTGCGTTCTACTTGC | [2] |
| *S. sanguinis* | GCATACTATGGTTAAGCCACAGCC | [3] |

**Table S2.** Oligonucleotide primers used in quantitative PCR for microbial quantification

| Bacterial species | Sequence (5´- 3´) | References |
| --- | --- | --- |
| *S. mutans* | F-GCCTACAGCTCAGAGATGCTATTCT  R-GCCATACACCACTCATGAATTGA | [4] |
| *S. gordonii* | F-GCTTGCTACACCATAGACT  R-CCGTTACCTCACCTACTAG | [5] |
| *S. sanguinis* | F-AGTTGCCATCATTGAGTTG  R-GTACCAGCCATTGTAACAC | [5] |


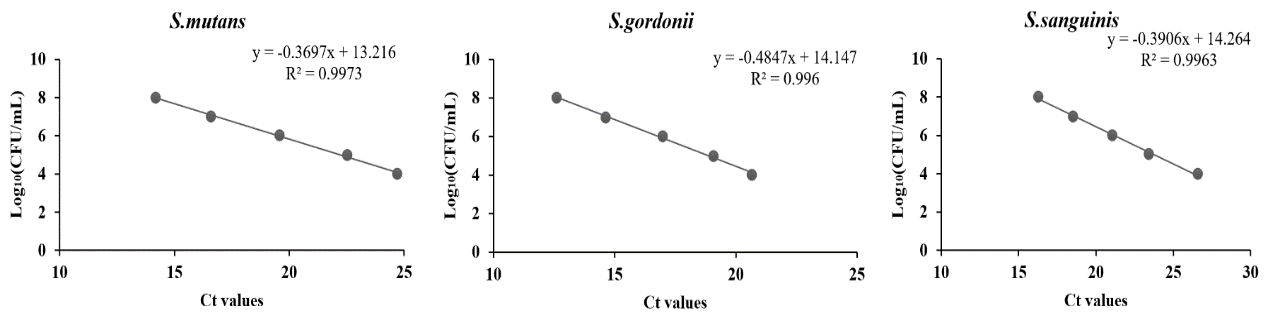


**Figure S1.** Standard curves for species-specific qPCR as plotted by known concentration of DNA of *S. mutans*, *S. gordonii* or *S. sanguinis*.

**References**

[1] Paster BJ, Bartoszyk IM, Dewhirst FE. Identification of oral streptococci using PCR-based, reverse-capture, checkerboard hybridization. Methods in Cell Science. 1998;20:223-31.

[2] Zhang K, Wang S, Zhou X, Xu HH, Weir MD, Ge Y, et al. Effect of antibacterial dental adhesive on multispecies biofilms formation. J Dent Res. 2015;94:622-9.

[3] Zheng X, Zhang K, Zhou X, Liu C, Li M, Li Y, et al. Involvement of gshAB in the interspecies competition within oral biofilm. J Dent Res. 2013;92:819-24.

[4] Yoshida A, Suzuki N, Nakano Y, Kawada M, Oho T, Koga T. Development of a 5' nuclease-based real-time PCR assay for quantitative detection of cariogenic dental pathogens Streptococcus mutans and Streptococcus sobrinus. J Clin Microbiol. 2003;41:4438-41.

[5] Zhou Y, Yang J, Zhang L, Zhou X, Cisar JO, Palmer RJ, Jr. Differential Utilization of Basic Proline-Rich Glycoproteins during Growth of Oral Bacteria in Saliva. Appl Environ Microbiol. 2016;82:5249-58.
